# Can structured integration of BI-RADS criteria by a clinical decision rule reduce the number of unnecessary biopsies in BI-RADS 4 lesions? A systematic review and meta-analysis

**DOI:** 10.1007/s00330-024-11274-6

**Published:** 2024-12-18

**Authors:** Giulia Vatteroni, Matthias Dietzel, Pascal A. T. Baltzer

**Affiliations:** 1 Department of Biomedical Sciences, Humanitas University, Via R. Levi Montalcini 4, 20072 Milan, Pieve Emanuele Italy; 2 Department of Radiology, University Hospital Erlangen, Erlangen, Germany; 3 Department of Biomedical Imaging and Image-guided Therapy, Division of General and Pediatric Radiology, Medical University of Vienna and General Hospital, Waehringer Guertel 18-20, 1090 Vienna, Austria

**Keywords:** Clinical decision rule, Breast, Sensitivity and specificity, Evidence-based medicine, Imaging

## Abstract

**Aim:**

This systematic review and meta-analysis investigate the added value of structured integration of Breast Imaging Reporting and Data System (BI-RADS) criteria using the Kaiser score (KS) to avoid unnecessary biopsies in BI-RADS 4 lesions.

**Material and methods:**

A systematic review and meta-analysis were conducted using predefined criteria. Eligible articles, published in English until May 2024, dealt with KS in the context of BI-RADS 4 MRI. Two reviewers extracted study characteristics, including true positives (TP), false positives (FP), true negatives (TN), and false negatives (FN). Sensitivity, specificity, negative likelihood ratio, and positive likelihood ratio were calculated using bivariate random effects. Fagan nomograms identified the maximum pre-test probability at which post-test probabilities of a negative MRI aligned with the 2% malignancy rate benchmark for downgrading BI-RADS 4 to BI-RADS 3. *I*² statistics and meta-regression explored sources of heterogeneity. *p*-values < 0.05 were considered significant.

**Results:**

Seven studies with 1877 lesions (833 malignant, 1044 benign) were included. The average breast cancer prevalence was 47.3%. Pooled sensitivity was 94.3% (95%-CI 88.9%–97.1%), and pooled specificity was 68.1% (95%-CI 56.6%–77.7%) using a random effects model. Overall, 52/833 cases were FNs (6.2%). Fagan nomograms showed that KS could rule out breast cancer in BI-RADS 4 lesions at a pre-test probability of 20.3% for all lesions, 25.4% for masses, and 15.2% for non-mass lesions.

**Conclusions:**

In MRI-assessed BI-RADS 4 lesions, applying structured BI-RADS criteria with the KS reduces unnecessary biopsies by 70% with a 6.2% FN rate. Breast cancer can be ruled out up to pre-test probabilities of 20.3%.

**Key Points:**

***Question*** *What, if any, value is added by structured integration of BI-RADS criteria using the Kaiser Score (KS) to avoid unnecessary biopsies in BI-RADS 4 lesions?*

***Findings*** *The structured integration of BI-RADS criteria using the Kaiser Score (KS) reduces unnecessary biopsies in BI-RADS 4 lesions by 70%.*

***Clinical relevance*** *The structured approach offered by the Kaiser Score (KS) avoids unnecessary recalls, potentially reducing patient anxiety, lessening the burden on medical personnel, and the need for further imaging and biopsies due to more objective and thus efficient clinical decision-making in evaluating BI-RADS 4 lesions.*

## Introduction

Breast MRI is the most sensitive tool for breast cancer diagnosis, routinely applied for pre-operative staging, neoadjuvant therapy monitoring, high-risk screening, and management of equivocal findings on mammography or ultrasound [1, 2] where performing MRI can avoid unnecessary follow-up or biopsies due to its high negative predictive value [3–7].

Breast MRI is usually reported according to the Breast Imaging Reporting and Data System (BI-RADS) lexicon, a standardized reporting tool used worldwide, which deals with many aspects, from the examination technique to the management recommendation. BI-RADS does not provide a clinical decision rule that combines diagnostic criteria in a structured manner [6]. Such a lack, however, makes breast MRI assessment challenging, subjective, and experience-dependent [8–10]. As breast imagers are trained not to miss cancer, the threshold to call a lesion BI-RADS 4 and recommend biopsies is low [11].

The Kaiser score (KS) is a machine-learning-derived and independently validated decision algorithm designed to facilitate the assessment of breast lesions in MRI [6]. It forces a structured and integrative review of BI-RADS criteria, providing a systematic approach to breast MRI evaluation. First introduced in 2013 [12] and commonly referred to as the “Kaiser score” since 2018 [13], the KS is a classification tree organized into three levels, incorporating five diagnostic BI-RADS criteria: lesion margins (including the presence of speculations), enhancement curve type, internal enhancement pattern, and presence of edema [12, 14]. The remaining BI-RADS criteria did not contain additional diagnostic information and were thus excluded from the decision algorithm.

By combining the diagnostic independent criteria, the KS assigns a value ranging from 1, indicating the lowest risk of breast cancer, to 11, indicating the highest risk. The score can be then translated into BI-RADS categories: KS 1–4 corresponds to BI-RADS 2/3, KS 5–7 to BI-RADS 4, and KS 8–11 to BI-RADS 5. A KS threshold above 4 is commonly used to recommend a biopsy [13, 15].

Consequently, the KS approach supports the structured integration of BI-RADS criteria for stratifying the risk of breast cancer in breast MRI lesions.

Empirical evidence supporting the use of the KS to reduce unnecessary biopsy rates remains limited. This systematic review and meta-analysis aim to investigate the added value of the structured integration of BI-RADS criteria using the KS to avoid unnecessary biopsies in BI-RADS 4 lesions.

## Methods

### Study design and eligibility criteria for study selection

This is a systematic review and meta-analysis of published research data on the rate of potentially avoidable needle biopsies of MRI suspicious findings if KS is applied to contrast-enhanced MRI. The research strategy was defined before the start of the study and the analysis was performed in adherence with the Preferred Reporting Items for Systematic Reviews and Meta-Analyses (PRISMA) [16]. Each eligible article had to provide at least raw data from which to extract true-positive (TP), false-positive (FP), true-negative (TN), and false-negative (FN) findings. A reference standard had to be established—either histopathologic examination or imaging follow-up of at least 12 months a finding as benign or malignant. Studies focusing on KS not dealing with BI-RADS 4 lesions and without comparison with histology were considered not representative of the research question and excluded. The study by Cloete et al [17] published in 2018, even if it answered the research question was excluded since all the lesions were benign. We also excluded studies with incomplete data, meta-analyses and reviews, comments, articles not in English, and other non-related studies.

### Search strategy

A computerized query and systematic review of articles in PubMed was performed by a reader (G.V. with more than 6 years of experience in breast imaging). All articles listed online up to April 23, 2024, with no lower timepoint limit, were considered. The search term “Kaiser score breast” was used. For PubMed, the search string read “kaiser”(All Fields) AND (“score”(All Fields) OR “score s”(All Fields) OR “scored”(All Fields) OR “scores”(All Fields) OR “scoring”(All Fields) OR “scorings”(All Fields) AND (“breast”(MeSH Terms) OR “breast”(All Fields) OR “breasts”(All Fields) OR “breast s”(All Fields). Moreover, since this clinical decision rule was previously referred to as the “Tree flowchart” and that was named the “Kaiser score” only in 2018 [13], papers citing the article that introduced the algorithm initially in 2013 [12] were scanned on Google Scholar for additional eligible studies (“forward snowballing”).

Consequently, the titles and abstracts of the search results were reviewed for eligibility, and the results were exported to an Excel spreadsheet. Full texts of eligible studies were retrieved and critically reviewed with a second reader (P.A.T.B., with 21 years of experience in breast imaging), and discrepancies were resolved by consensus.

### Data collection and risk of bias

Two independent readers (G.V., P.A.T.B) selected eligible studies and extracted the data into a predefined Excel spreadsheet, and assessed the risk of bias and applicability concerns according to Quality Assessment of Diagnostic Accuracy Studies (QUADAS)-2 [18].

The extracted data included: author, year, journal, paper title, setting, design (prospective and retrospective), indication for MRI imaging, reference standard, number of patients, number of Mass lesions and Non-mass lesions, number of benign and malignant lesions, MRI field strength, MRI Vendor, coil, contrast agent, contrast dose, number of readers and inter-reader agreement assessment.

Extraction of imaging results (true positive, false positive, true negative, and false negative) was performed. “Positive” was defined as malignant, and if a study classified high-risk lesions as malignant, we re-classified it as benign for consistency. Borderline phyllodes, even if not strictly malignant, were considered positive since they principally require surgical excision. If the study involved more than one reader, the raw data results average was calculated and reported. As all included MRI lesions had a biopsy indication, all were considered final imaging assessment BI-RADS 4. If a study resulted as an outlier, authors were contacted to ask for additional information.

### QUADAS assessment

QUADAS assessment was performed in consensus by both readers, evaluating four domains for each study: patient selection, index test, reference standard, and flow and timing. Each domain was evaluated in terms of risk of bias, and the first 3 domains were also assessed in terms of concerns regarding applicability [18]. The risk of bias was assessed in regard to the study aims. Bias was rated according to present—a bias that would definitely alter the study results or applicability; absent—no bias that would alter study results or applicability; and unclear—lack of information that does not allow to rule in or rule out definite bias.

### Statistical analysis

All analyses were performed by using commercial (STATA/SE 15, StataCorp, MIDAS plugin) and open-source (OpenMeta Analyst 0.1, https://www.brown.edu/public-health/cesh/resources/software) software.

Pooled effect sizes were calculated using a bivariate random effects model of combined sensitivity and specificity, and a summary receiver operating characteristics (sROC) curve was calculated.

Sources of heterogeneity were investigated by using a random effects meta-regression, and the influence of year of publication, indication for examination, and technical set-up parameters on the diagnostic performance metrics of sensitivity, specificity, and diagnostic odds ratio was investigated. The inconsistency *I*^2^ was calculated and classified as low (<= 25%), medium (<= 50%), or high (<= 75%).

A Fagan nomogram was used to provide post-test probabilities based on variable pre-test probabilities for clinical decision-making. *p* < 0.05 were considered statistically significant.

## Results

**Fig. 1:**
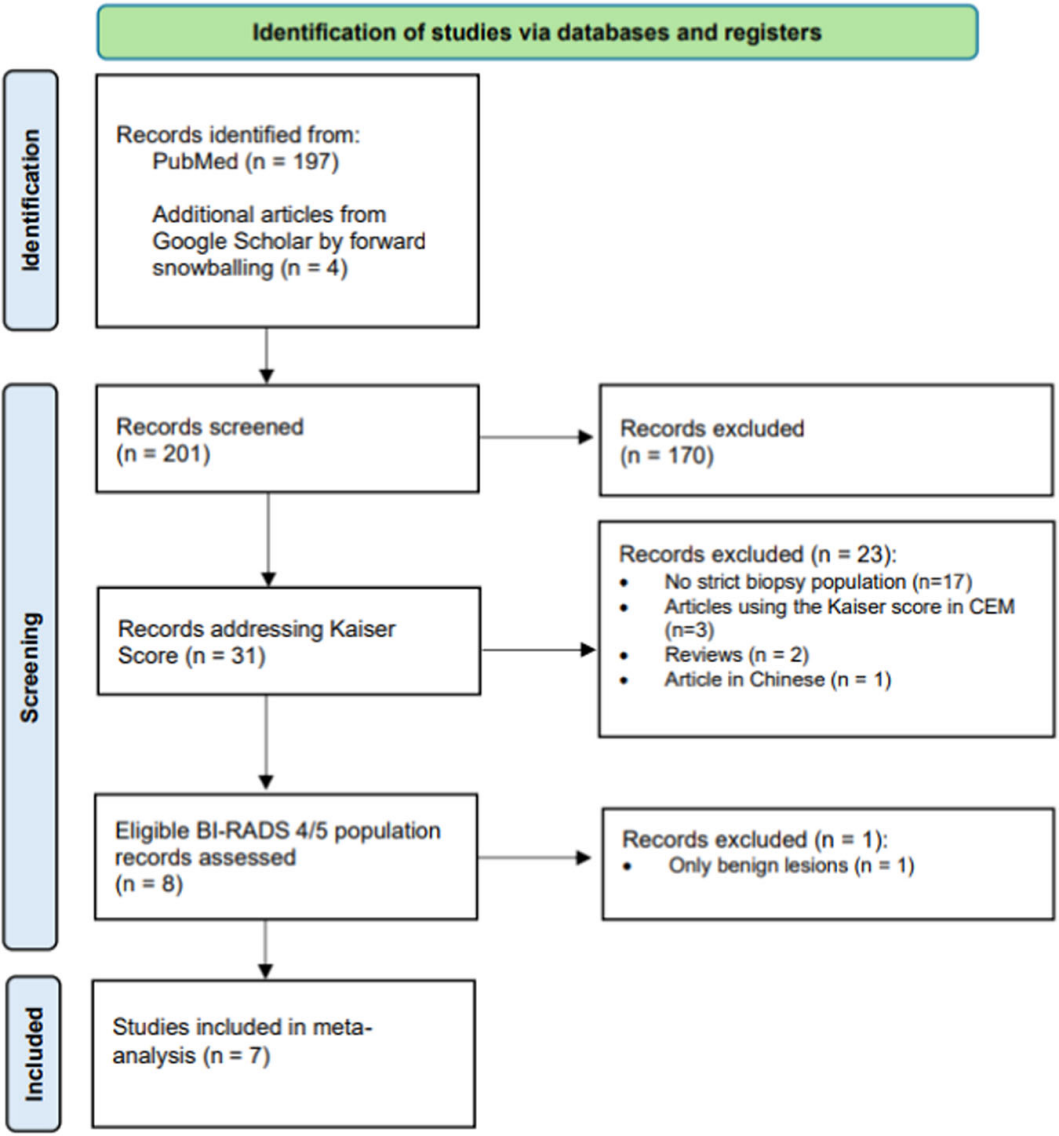
Preferred reporting items for systematic reviews and meta-analyses 2020 flowchart showing the systematic search results and the selection process towards the included studies. CEM, contrast-enhanced mammography

The study selection process is summarized in the PRISMA flowchart [16] (Fig. 1).

Seven studies, comprising 1877 lesions (833 malignant and 1044 benign), were eligible for data synthesis. Breast cancer prevalence ranged between 21% [19] and 69% [20], with an average of 47.3%. The study design was retrospective in all the studies. Patient recruitment was consecutive in six studies, while no information was available in one study [20]. All studies had histology workup of BI-RADS 4 lesions as a reference standard. Extracted data are summarized in Table 1.

**Table 1:** Key parameters extracted from the eligible studies

Author	Setting	MR indication	No of lesion/no of patient	Prevalence of malignancy (%)	TP	FP	TN	FN
Woitek et al [19]	BI-RADS 4 MRI	MRI-only lesions	469/454	21	79	65	306	19
Milos et al [25]	BI-RADS 4 MRI	High risk	183/159	22	39	54	88	2
Wengert et al [22]	BI-RADS 4 gen.	BI-RADS 4 Calcification	167/167	56	93	28	44	2
Meng et al [21]	BI-RADS 4 MRI	Mixed^a^	268/243	38	96	50	116	6
An et al [24]	BI-RADS 4 MRI	Mixed^b^	273/246	57	150	19	99	5
Jajodia et al [20]	BI-RADS 4 gen.	Problem-solving	316/310	69	209	54	47	6
Pan et al [23]	BI-RADS 4 MRI	Mixed^c^	201/194	63	115	29	45	12

*No* number, *NA* not available, *TP* true positive, *FP* false positives, *TN* true negatives, *FN* false negatives

^a^ Mixed indications, excluded all patients undergoing treatment, then all MR BI-RADS 2, 3 and excluded

^b^ Opportunistic screening, problem-solving for equivocal clinical or radiologic findings on mammography or ultrasound, post-operative follow-up, and evaluation response of chemotherapy

^c^ Problem-solving, pre-operative staging, or assessment of the response after therapy

One reader was involved in two studies [19, 21] in which a subset of lesion was read by a second reader to calculate the kappa agreement, two readers were involved in four studies [20, 22–24], and three readers were involved in one study [25]. In the multiple reader studies, independent reading was performed in four studies [22–25] while in one study readings were performed in consensus [20]. The readers were blinded to the histopathological results in all the studies.

The inter-reader agreement was assessed in all the studies except one [23]. Kappa agreement was almost perfect in four studies [19–21, 24], while fair to moderate in two studies [22, 25]. Extracted kappa are summarized in Table 2.

**Table 2:** Study characteristics

Author	Field of strength (T)	Vendor	Coil	Contrast agent	CM Dose (mmol/kg)	No of readers	*K* agreement
Woitek et al [19]	1.0–1.5-3	Philips Siemens Healthineers	Dedicated breast coil	Gadoterate meglumine, Gadobenate dimeglumine, Gadobutrol, Gadoteridol, Gadodiamide	0.1	1^a^	0.944
Milos et al [25]	1.0–1.5	Philips Siemens Healthineers	Dedicated breast coil	NA	NA	3	0.393–0.560
Wengert et al [22]	1.5–3	Siemens Healthineers	4-18-16 channel	NA	NA	2	0.510
Meng et al [21]	3	GE Healthcare	8 channel	Gadodiamide	0.1	1^a^	0.913
An et al [24]	3	Siemens Healthineers	8 channel	Gadopentatic acid	0.1	2	0.809
Jajodia et al [20]	1.5	Siemens Healthineers	Dedicated breast coil	Gadodiamide	0.1	2	0.812–0.883
Pan et al [23]	1.5-3	Philips Siemens Healthineers	8 channel	Gadopentatic acid	0.1	2	NA

*No* number, *NA* not available, *CM* contrast medium

^a^Subset read by two readers

All the studies used a dedicated breast coil and examined the patients in the prone position. Five studies used a multiparametric protocol including T2-weighted sequences, diffusion-weighted imaging, and dynamic contrast-enhanced imaging [20, 21, 23–25]. Woitek et al [19] did not include diffusion-weighted imaging, Wengert et al [22] used a multiparametric protocol in accordance with the European Society Of Breast Imaging (EUSOBI) and European Society of Breast Cancer Specialists (EUSOMA) recommendation [1, 26]. Study-specific characteristics are given in Table 2.

**Fig. 2:**
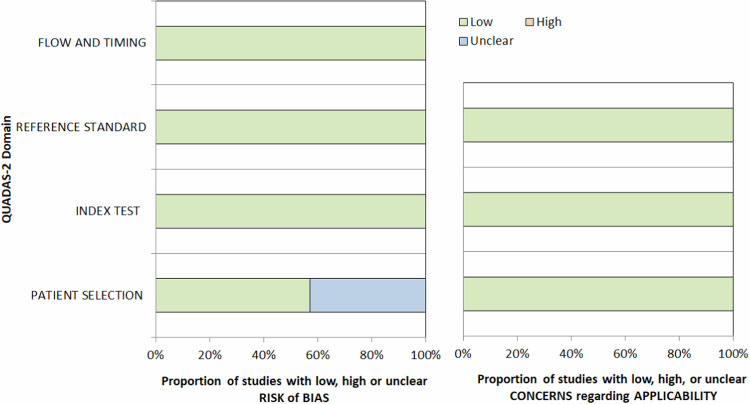
QUADAS-2 assessment. Results show a low risk of bias and no concerns regarding the applicability of the included studies

QUADAS assessment is summarized in Fig. 2. Regarding patients selection, it was unclear in 3 studies [20–22], while in the other studies there was a low risk of bias. Regarding index test, in one study [19] there was not a predefined KS threshold used, although it was considered at low risk of bias as the other studies. There were no concerns regarding the applicability of the included studies to the research questions.

**Fig. 3:**
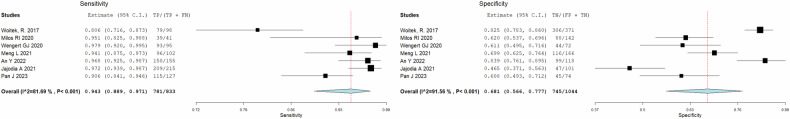
Forest plot of sensitivity and specificity data synthesis using a random effects model (Kaiser score readings)

**Fig. 4:**
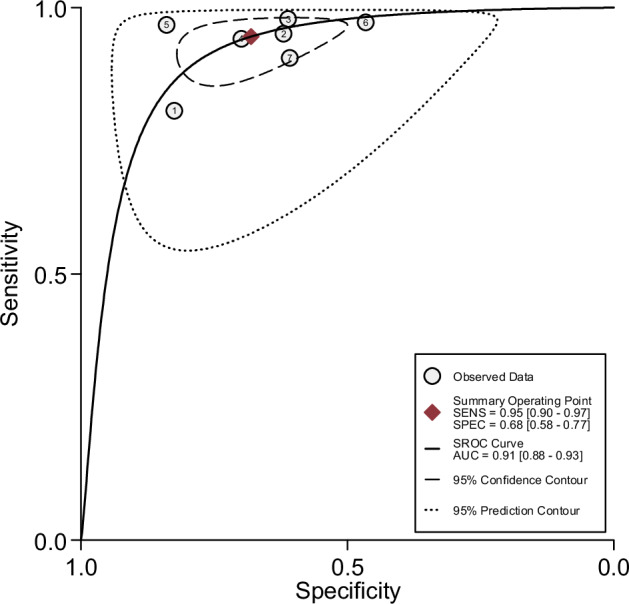
Summary ROC curve derived from bivariate modeling of sensitivity and specificity from individual studies utilizing the Kaiser score in BI-RADS 4 lesions

Considering all the lesions, pooled sensitivity was 94.3% (95%-CI 88.9%–97.1%) according to the random effect model and 95% (95%-CI 90.0%–97.0%) according to the bivariate model, ranging from 80.6% [19] to 97.9% [22]. Pooled specificity was 68.1% (95%-CI 56.6%–77.7%) calculated with the random effect model and 68.0% (95%-CI 58.0%–77.0%) according to the bivariate model. Specificity ranged from 46.5% [20] to 83.9% [24]. Random effect model and sROC curve derived from the bivariate model are shown in Figs. 3 and 4, respectively. All the studies but one [21] reported the number of Mass and Non-mass lesions. The six studies included in the sub-analysis comprise a total of 947 Masses (461 malignant and 486 benign) and 615 Non-masses (269 malignant and 346 benign). Extracted data on Masses and Non-masses are summarized in Table 3.

**Table 3:** Extracted data on Mass and Non-mass lesions

Author	Lesion type	No of lesions	Malignant	Benign	TP	FP	TN	FN
Woitek et al [19]	Mass	270	68	202	59	44	158	9
	NME	199	30	169	20	21	148	10
Milos et al [25]	Mass	88	24	64	24	29	35	0
	NME	48	10	38	9	17	21	1
Wengert et al [22]	Mass	51	29	22	29	7	15	0
	NME	116	66	50	64	20	30	2
Meng et al [21]	Mass	NA	NA	NA	NA	NA	NA	NA
	NME	NA	NA	NA	NA	NA	NA	NA
An et al [24]	MASS	214	111	103	107	12	91	4
	NME	59	44	15	43	7	8	1
Jajodia et al [20]	Mass	183	137	46	132	25	22	4
	NME	133	84	49	77	29	25	2
Pan et al [23]	Mass	141	92	49	86	19	30	6
	NME	60	35	25	29	10	15	6

*NME* non-mass enhancement, No number, *TP* true positive, FP false positives, *TN* true negatives, *FN* false negatives, *NA* not available

Analyzing Mass lesions, pooled sensitivity was 94.7% (95%-CI 90.1%–97.3%) (random effect model), ranging from 86.8% [19] to 98.3% [22], while pooled specificity was 68.1% (95%-CI 53.3%–80.0%) (random effect model), ranging from 46.8% [20] to 88.3% [24] (Supplemental Fig. 1).

Looking only at Non-mass lesions, pooled sensitivity was 91.9% (95%-CI 78.5%–97.3%) (random effect model), ranging from 66.7% [19] and 97.7% [24], while pooled specificity was 62.6% (95%-CI 44.1%–78.0%) (random effect model), ranging from 46.3% [20] and 87.6% [19] (Supplemental Fig. 2).

Pooled negative likelihood ratio was 0.08 (95%-CI 0.049–0.128) for all the lesions, 0.060 (95%-CI 0.034–0.107) for Masses, and 0.114 (0.056–0.235) for Non-masses.

Pooled positive likelihood ratio was 2.966 (95%-CI 2.224–3.956) for all the lesions, 3.031 (95%-CI 2.074–4.429) for Masses, and 2.389 (95%-CI 1.778–3.211) for Non-mass lesions.

### Sources of heterogeneity

There was statistically significant (*p* < 0.001) high heterogeneity (*I*^2^ = 81.7% for sensitivity and *I*^2^ = 91.6% for specificity) between the included studies.

Meta-regression revealed “year of publication” as a factor influencing the diagnostic performance of KS even though this did not prove statistical significance (*p* = 0.065, see supplemental Fig. 3). This finding was mainly due to the low sensitivity in the Woitek study [19]. Upon contacting the authors, we found that the heterogenous MRI protocols employed did regularly rely on abbreviated dynamic protocols with sometimes only two post-contrast repetitions that can hamper curve-type assessment. Neither indication for the examination nor technical setup parameters did influence diagnostic parameters upon meta-regression (*p* > 0.05, each).

### Details of false-negative findings among invasive cancers and all malignant lesions

All the studies provided the number and histology of missed cancers. All but one study [21] reported the lesion type. All FN details are reported in Table 4.

**Table 4:** Radiological and pathological data of false negatives

Author	No. of malignant lesions	No. of FN	Lesion type	Not invasive FN	Pathology	Invasive FN	Pathology
Woitek et al [19]	98	19	9 Mass 10 NME	9	DCIS	10	8 IDC 1 ILC 1 Mucinous
Milos et al [25]	41	4 (2^a^)	2 NME 2 Foci	1	DCIS	3	3 Luminal A
Wengert et al [22]	95	4 (2^a^)	4 NME	3	DCIS	1	1 IDC G2 ER + PR+
Meng et al [21]	102	6	N.A.	1	DCIS	5	1 IC 3 Mucinous 1 Medullary
An et al [24]	155	5	4 Mass 1 NME	3	1 DCIS 2 Phylloides	2	1 Mucinous Ca 1 Adenoid cystic Ca
Jajodia et al [20]	215	6	4 Mass 2 NME	4	NA	2	2 Triple negative
Pan et al [23]	127	12	6 Mass 6 NME	8	7 DCIS 1 intraductal Papillary Ca	4	3 IDC 1 Mucinous Ca

*No*. number, *FN* false negatives, Ca carcinoma, *DCIS* ductal carcinoma in situ, *NA* not available, *IDC* invasive ductal carcinoma, *IC* invasive cancer, *ILC* invasive lobular carcinoma, *ER* estrogen receptor, *PR* progesterone receptor, *NME* non-mass enhancement, *NA* not available

^a^ Average values were taken for quantitative data synthesis while all cases are listed here

### Pre-test probability of KS for ruling out malignancy (invasive cancer and DCIS)

**Fig. 5:**
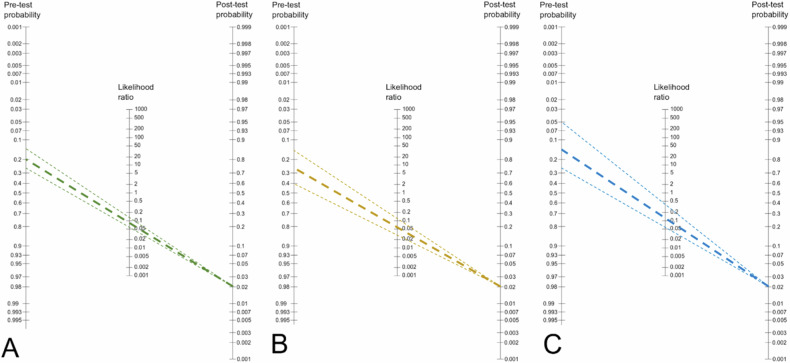
Fagan nomograms based on pooled (random effects modeling) negative likelihood ratios for all lesions (**A**), mass lesions (**B**), and non-mass lesions (**C**). Thick lines represent the pooled summary estimate, while the thin lines represent its 95% CI. The lines focus on the 2% post-test probability benchmark for BI-RADS 3. The left part of the nomogram thus depicts the pre-test probability range up to which a negative structured BI-RADS assessment using the Kaiser score can downgrade BI-RADS 4 lesions to BI-RADS 3 to potentially avoid biopsy

To estimate the ability of KS to rule out cancer in BI-RADS 4 lesions, we applied the pooled negative likelihood ratios to a Fagan nomogram (Fig. 5). Up to a pre-test probability of 20.3% the negative likelihood ratio (0.08) of applying the KS results in a post-test probability of 2%, allowing to downgrade the lesion while meeting BI-RADS benchmarks.

The same goal was achieved up to a pre-test probability of 25.4% (LR-0.06) in masses and 15.2% (LR-0.114) in non-masses.

## Discussion

This systematic review and meta-analysis demonstrate the potential of the structured assessment of BI-RADS criteria within the framework of the KS to significantly reduce unnecessary biopsies. Out of 10 lesions that would have undergone biopsy with a conventional workup, 6 to 8 biopsies could be avoided using this structured approach.

The BI-RADS lexicon was introduced in radiology as the first standardized reporting method, becoming an established part of most radiology subspecialties [27, 28]. From its introduction, numerous reporting systems followed its example, e.g. the PIRADS for prostate [29]. However, the BI-RADS does not provide the reader a clinical decision tool as the PIRADS does. Without a clear clinical decision rule, diagnosis is unstructured, subjective and dependent on radiologists’ experience, making reporting MRI often difficult.

The KS forces structured BI-RADS assessment integrating diagnostic BI-RADS features into an imaging phenotype associated with a probability of malignancy.

Distinguishing between benign and malignant lesions in MRI is indeed challenging, leading to benign results in 40.2–84.6% of BI-RADS 4 or 5 findings [12, 30]. While biopsies are minimally invasive and safe, their associated costs, personnel requirements, and psychological impact on patients make avoiding false-positive biopsies desirable [31].

Our analysis shows that the KS provides a clinical advantage in evaluating BI-RADS 4 lesions, with a pooled specificity of 68.1%, maintaining high sensitivity and demonstrating that a clinical decision rule helps overcome subjective image-interpretation difficulties, allowing accurate and objective diagnosis, with high inter-reader agreement [19, 21].

Increasing specificity to avoid unnecessary biopsies may, on the other hand, decrease sensitivity, resulting in missed cancers. The overall false-negative rate in this study was 6%, exceeding the 2% benchmark for BI-RADS 3 categorization. However, this rate decreases to 3.2% when we consider only missed invasive cancers. In the analyzed studies the majority of cancers not diagnosed by MRI were DCIS or biologically not aggressive carcinomas. MRI is, in fact, more sensitive to identify clinically significant cancers [32] and, due to this reason, it could, therefore, be relatively safe to assume that downgrading a lesion would not change the patients’ prognosis but rather lead to a delayed diagnosis in a biologically less significant malignancy. This aspect, however, requires dedicated investigation, including follow-up exams [25].

We, therefore, used a Fagan nomogram to clarify whether the KS could be applied to rule out malignancy in the BI-RADS 4 category, which comprises a broad range of likelihood of malignancy (from 3% to 95%) [27]. According to our results, a negative MRI could downgrade BI-RADS 4 lesions with a pre-test probability of up to 20%. Its performance rises up to 25% in masses while decreasing to 15% in non-mass lesions. This practically means that, if applying the KS, a negative MRI could safely downgrade both masses and non-mass lesions classified as BI-RADS 4a, as well as some mass lesions within the BI-RADS 4b category. Consequently, non-mass enhancements must be interpreted with caution. The MRI BI-RADS lexicon lacks diagnostically specific non-mass descriptors. The KS applies criteria such as margins which are in the current BI-RADS reserved for mass lesions only. Readers not experienced with the KS may thus have difficulties in applying these criteria in the more subtle non-mass enhancements [12–14]. In four studies included in our analysis [19, 20, 22, 25], FN presented mainly as small lesions with a diameter of less than 8 mm. Margins and internal lesion enhancement assessment are challenging in these cases, particularly if image quality is low [19, 25, 33, 34].

Two studies reported a lower sensitivity [19, 23]. The lower sensitivity in the first [19] could be potentially related to the part use of “abbreviated protocols” comprising only two post-contrast sequences. This does affect curve type assessment leading to misinterpretation, as curve type is the second most important diagnostic criterion within the KS.

In the second study [23], the added value of microcalcifications in KS assessment of BI-RADS 4 lesions was evaluated. FN was mainly DCIS that is diagnosed in up to 90% mainly due to mammographic calcifications [35, 36].

Semantic criteria as those in the BI-RADS and in the KS are prone to a certain level of subjectivity and require thorough definition [8, 14]. To minimize subjectivity in KS assessment, the concept of a “default category” was introduced in 2022, meaning that a diagnostic criterion should only be evaluated as positive if it is present without doubt [6]. This novel concept however has not yet been backed up by empirical evidence.

Our study had limitations. The number of available studies was low, and high between-study heterogeneity limits the clinical applicability of pooled summary estimates. Additionally, as pointed out by Grippo et al [37] the KS diagnostic performance is significantly influenced by curve types, and correct initial enhancement timepoint determination is crucial to avoid FNs. For this reason, potential mistakes in correctly evaluating the initial time points could have occurred in the studies included. These points stress that the results of this study may underestimate the full potential of structured integration of BI-RADS criteria using the KS and should thus only be applied with care.

In the systematically reviewed literature, we noted some research gaps. First, studies generally refer to BI-RADS 4 MRI lesions without focusing on lesion characteristics on mammography or ultrasound. Since the KS is not a fully automatic algorithm, the radiologist can derive management recommendations considering each individual situation [6]. As first pointed out by Thomassin-Naggara et al [38], combining conventional, clinical, and MRI features could improve lesion assessment. As studies were retrospective, all potential benefits and harms of the KS remain theoretical. Prospective research may be required not only to assess harms and benefits in clinical application but also to establish appropriate follow-up intervals after downgrading BI-RADS 4 lesions. Of note, patient trust and acceptance are important and can only be assessed in real life clinical practice.

In the end, even if a diagnostic test has high accuracy, its application value in clinical practice matters. An individual risk assessment based on clinical, conventional imaging and KS features is required to solve the question: “Can I safely send this patient to follow-up without the risk of missing potentially life-threatening disease?”.

## Summary and conclusion

In conclusion, this systematic review and meta-analysis endorses the value of structured assessment of BI-RADS criteria. Compared to conventional assessment without a decision rule, this approach can significantly reduce the number of unnecessary biopsies by up to 70%. Further large-scale, setting-specific prospective studies are warranted to discuss the impact of false-negative findings on clinical management and patient outcomes.

## Supplementary information

ELECTRONIC SUPPLEMENTARY MATERIAL

## Abbreviations

BI-RADS: Breast Imaging Reporting and Data System
FN: False negatives
FP: False positives
KS: Kaiser score
PRISMA: Preferred Reporting Items for Systematic Reviews and Meta-Analyses
QUADAS: Quality Assessment of Diagnostic Accuracy Studies
sROC: Summary receiver operating characteristics
TN: True negatives
TP: True positives
