# Supplementary material for: Can structured integration of BI-RADS criteria by a clinical decision rule reduce the number of unnecessary biopsies in BI-RADS 4 lesions? A systematic review and meta-analysis
Source: Eur Radiol. 2024 Dec 18;35(3):1504–13. doi: 10.1007/s00330-024-11274-6 (PMC11836227; doi:10.1007/s00330-024-11274-6)
Supplement: Supplementary file 1 — ELECTRONIC SUPPLEMENTARY MATERIAL [file 330_2024_11274_MOESM1_ESM.pdf]

Can structured integration of BI-RADS criteria by a clinical decision rule reduce the number of Unnecessary Biopsies in BI-RADS 4 Lesions? A Systematic Review and Meta-Analysis

ELECTRONIC SUPPLEMENTARY MATERIAL

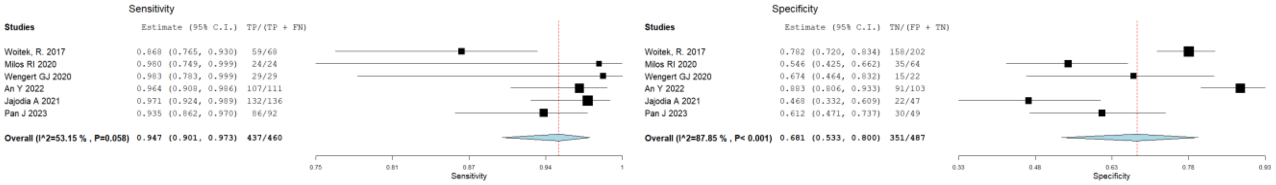

Supplemental Figure 1:

Forest plot of sensitivity and specificity data synthesis using a random effects model (Kaiser score readings) in mass lesions.

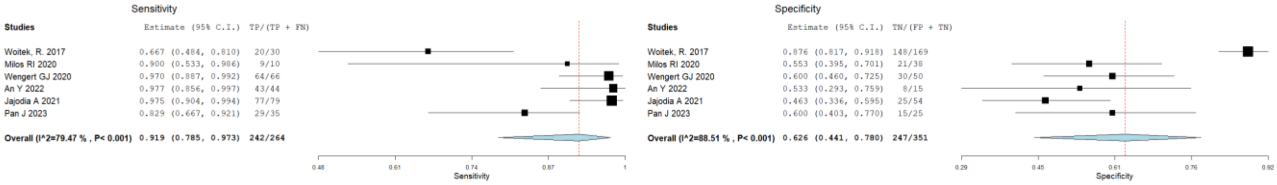

Supplemental Figure 2:

Forest plot of sensitivity and specificity data synthesis using a random effects model (Kaiser score readings) in non-mass lesions.

Eur Radiol (2024) Vatteroni G, Dietzel M, Baltzer PAT.

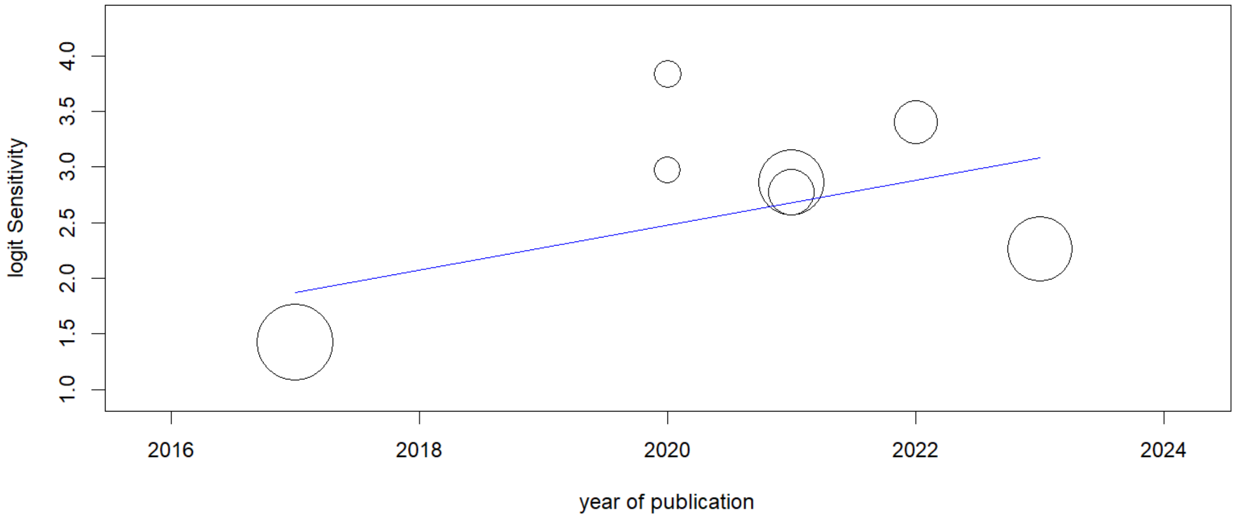

Supplemental Figure 3:

Meta-regression results showing a trend towards higher sensitivity the later a paper was published. This effect did not reach statistical significance (P=0.065)
